# Ensemble encoding of nociceptive stimulus intensity in the rat medial and lateral pain systems

**DOI:** 10.1186/1744-8069-7-64

**Published:** 2011-08-24

**Authors:** Yang Zhang, Ning Wang, Jin-Yan Wang, Jing-Yu Chang, Donald J Woodward, Fei Luo

**Affiliations:** 1 Neuroscience Research Institute and Department of Neurobiology, Peking University Health Science Center, Beijing, China; 2 Key Laboratory of Mental Health, Institute of Psychology, Chinese Academy of Sciences, Beijing, China; 3 Neuroscience Research Institute of North Carolina, Winston-Salem, NC, USA

**Keywords:** Laser, Intensity, Pain, Single unit, Ensemble encoding

## Abstract

**Background:**

The ability to encode noxious stimulus intensity is essential for the neural processing of pain perception. It is well accepted that the intensity information is transmitted within both sensory and affective pathways. However, it remains unclear what the encoding patterns are in the thalamocortical brain regions, and whether the dual pain systems share similar responsibility in intensity coding.

**Results:**

Multichannel single-unit recordings were used to investigate the activity of individual neurons and neuronal ensembles in the rat brain following the application of noxious laser stimuli of increasing intensity to the hindpaw. Four brain regions were monitored, including two within the lateral sensory pain pathway, namely, the ventral posterior lateral thalamic nuclei and the primary somatosensory cortex, and two in the medial pathway, namely, the medial dorsal thalamic nuclei and the anterior cingulate cortex. Neuron number, firing rate, and ensemble spike count codings were examined in this study. Our results showed that the noxious laser stimulation evoked double-peak responses in all recorded brain regions. Significant correlations were found between the laser intensity and the number of responsive neurons, the firing rates, as well as the mass spike counts (MSCs). MSC coding was generally more efficient than the other two methods. Moreover, the coding capacities of neurons in the two pathways were comparable.

**Conclusion:**

This study demonstrated the collective contribution of medial and lateral pathway neurons to the noxious intensity coding. Additionally, we provide evidence that ensemble spike count may be the most reliable method for coding pain intensity in the brain.

## Background

The ability to evaluate the intensity of painful stimuli is a major property of the nociceptive system. Multiple studies have shown that discrimination of nociceptive stimulus intensity is the function of the lateral pain pathway, which participates in the sensory discrimination of pain [1-3]. Kenshalo et al. concluded that nociceptive neurons in the primary somatosensory cortex (SI) are involved in the encoding process by which monkeys perceive the intensity of noxious thermal stimulation [1]. Apkarian and Shi found that the proportion of responsive neurons in the lateral thalamus of squirrel monkey was increased with the noxious stimulus intensity [4].

In recent years, more and more studies accepted that the medial pain pathway could also have the ability to encoding the noxious stimulus intensity [5]. Coghill et al. employed a fMRI study of human subjects and suggested that a highly distributed, bilateral supraspinal mechanism engaged in the processing of pain intensity, including the brain areas in both the lateral and medial pain pathways, such as somatosensory cortex, anterior cingulate cortex (ACC) and the dorsomedial and centromedian thalamus [6]. Buchel et al. also provided evidence using human fMRI that the ACC have a role in coding pain intensity [7]. In an electrophysiological study of rat primary sensorimotor cortex (SmI) and ACC, Kuo and Yen have investigated the single-unit responses and found a noxious intensity-related activation in the recorded areas [8].

Although many studies have proved the ability of noxious intensity coding in the supraspinal pain circuits, it is still unclear about the neural encoding patterns in both pain pathways, and whether the coding ability of the medial pain system is comparable to that of the lateral pain system. In this study, a multiple single-unit recording technique for parallel recording of thalamo-cortical activity in the rat medial and lateral pain pathways was employed. Nociceptive responses were elicited in the hindpaw by laser pulses with progressively increasing intensity. Spike counts as well as several other commonly used coding schemes were computed to reveal the intensity coding strategy in the parallel pain pathways.

## Results

### Behavioral responses

**Figure 1:**
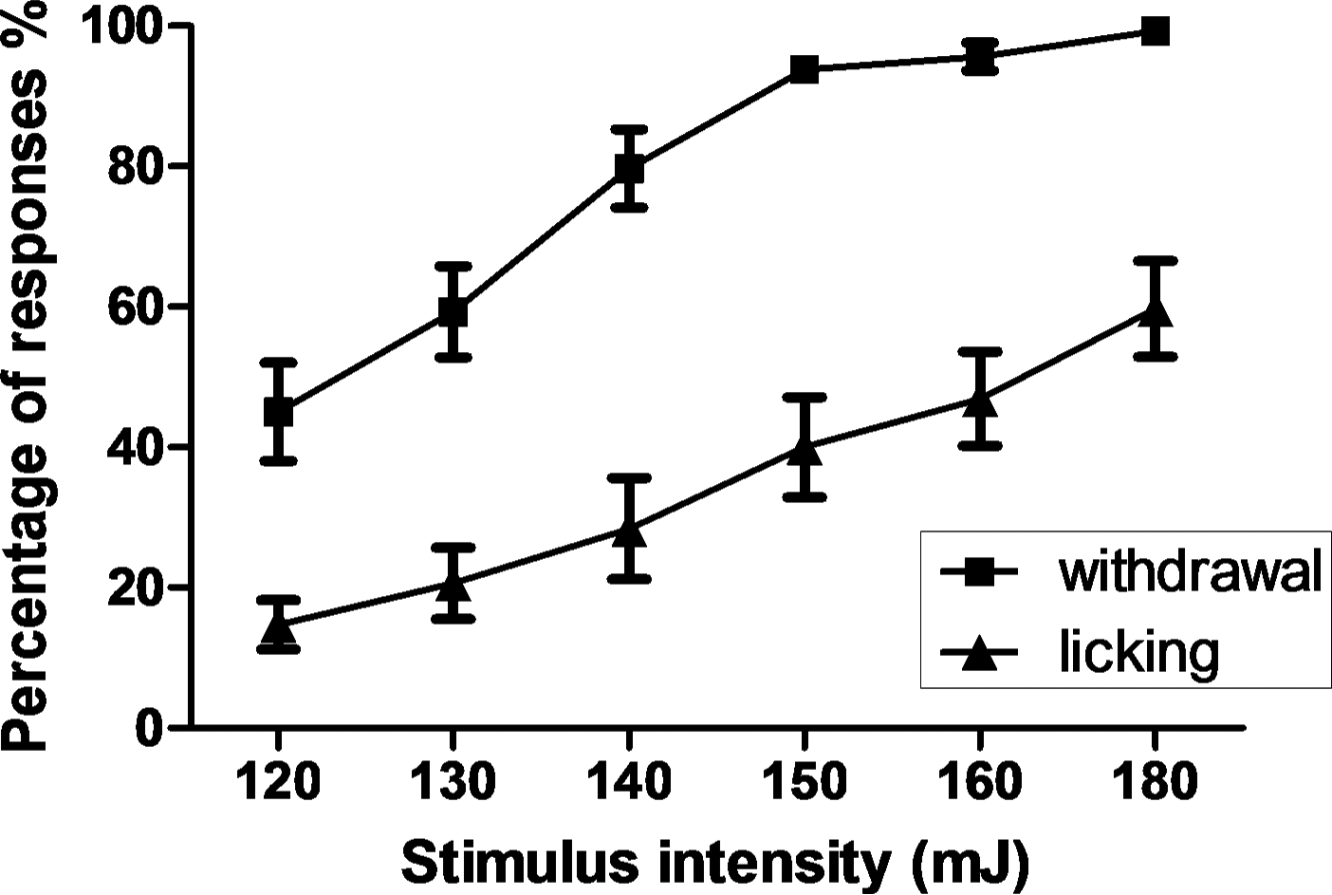
**The likelihood of observing paw withdrawal and paw licking behavioral responses increased with increasing laser stimulus intensity**. The percentage of behavioral responses was calculated as the number of stimulations producing the behavior/total number of stimulations × 100%. Data are presented as means ± SEM.

Noxious laser stimulation elicited obvious nocifensive behaviors, such as immediate withdrawal of the stimulated hind paw followed by licking. One-way ANOVAs revealed effects of stimulus intensity on paw withdrawal [F (5, 42) = 23.05, *p *< 0.0001] and paw licking [F (5, 42) = 7.458, *p *< 0.0001]. Graded behavioral responses were observed, with greater responses to higher intensity of laser stimuli (Figure 1). Linear trend analysis indicated significant increasing trends in response frequency with increasing stimulus intensity for both withdrawal and licking behaviors [*r^2 ^*= 0.6648, *p *< 0.0001; *r^2 ^*= 0.4645, *p *< 0.0001, respectively].

### General neuronal responses

**Figure 2:**
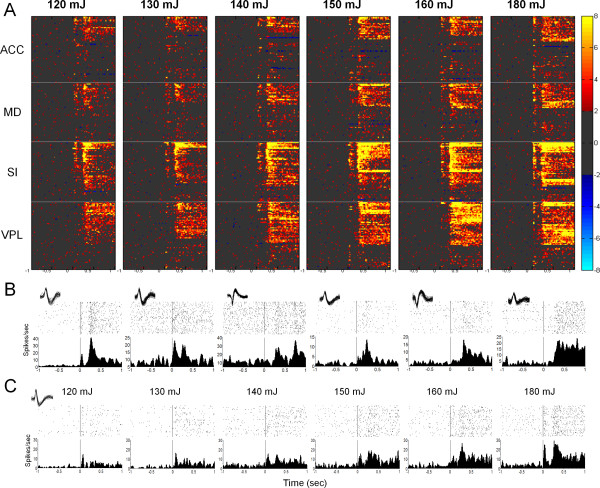
**Neuronal responses in the medial (ACC, MD) and lateral (SI, VPL) pain pathways induced by laser stimulation**. (A) Cluster plot depicting the temporal distribution patterns of neuronal activity in response to laser stimulation. The firing rates were transferred into Z-scores and neurons with Z-scores > 2 were accepted as significantly excited and < -2 as significantly inhibited. The colorbar legend ranges from light yellow for highest Z-score to light blue for lowest Z-score. Each line in the image represents the normalized activity of one neuron. (B) Raster and peri-event histograms showing various types of neuronal responses in the ACC, MD, SI, and VPL. Each dot in the raster (top) depicts the time of occurrence of a neuronal spike, with each row representing an individual trial. The peri-event histogram (bottom) illustrates the average firing rate of a neuron relative to the laser stimulation. Time = 0 on the *x *axis corresponds to the the laser stimulation onset. Insets show spike waveforms from each recording. (C) A representative example of an ACC neuron whose activity increased with stimulus intensity (120-180 mJ).

A total of 229 single neurons (61 ACC, 53 MD, 54 SI, and 61 VPL) were detected in eight male Sprague-Dawley rats. During the delivery of brief laser pulses, the responses were predominantly excitatory. Inhibitory responses were seldom encountered. On average, the laser stimuli evoked excitatory responses in no less than one-third of all recorded neurons within each brain region. As depicted in Figure 2A, the number of the responsive neurons and their firing rates increased with the intensity of the stimulus. The laser stimulus evoked various types of single-unit responses, including single-peak, double-peak, brief, and long-lasting activity with different peak latencies (see Figure 2B). Figure 2C illustrates an example of neurons in the ACC showing increasing neural activity with stimulus intensity.

**Figure 3:**
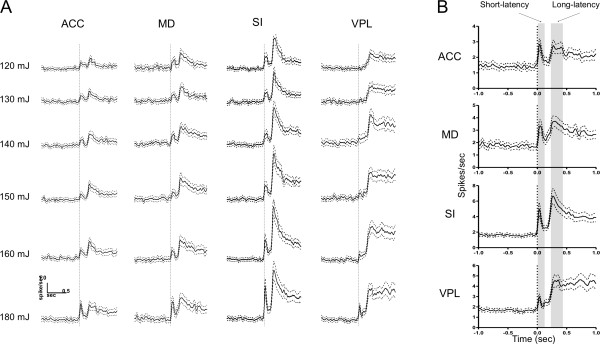
**The averaged single-unit response to laser stimulation for each brain region of interest at differing stimulation intensity levels (A) and averaged for all intensity levels (B)**. (A) Mean firing rate increased with stimulus intensity (120-180 mJ) for all the recorded regions (ACC, MD, SI, and VPL). Each panel represents the averaged firing rate of neurons around the time of laser stimulation (1.0 s pre- to 1.0 s post-stimulus). The dashed lines indicate 99% confidence limits. (B) When the single-unit responses are pooled across all the intensity levels (120-180 mJ), a double-peak response can be seen in all the recorded regions with a short-latency peak of less than 100 ms and a long-latency of 300-500 ms.

The average firing rates of multiple single neurons in each brain region are shown in Figure 3A. Mean firing rates generally increased with laser stimulus intensity. Double-peak responses were seen in all of the four examined brain regions, with short-latency (< 100 ms) and long-latency (300-500 ms) peaks. Figure 3B shows, for each region, the firing data averaged across all intensity sessions. The laser evoked obvious double-peak responses [9].

### Intensity coding at the single neuron level

#### Coding by number of active neurons

**Figure 4:**
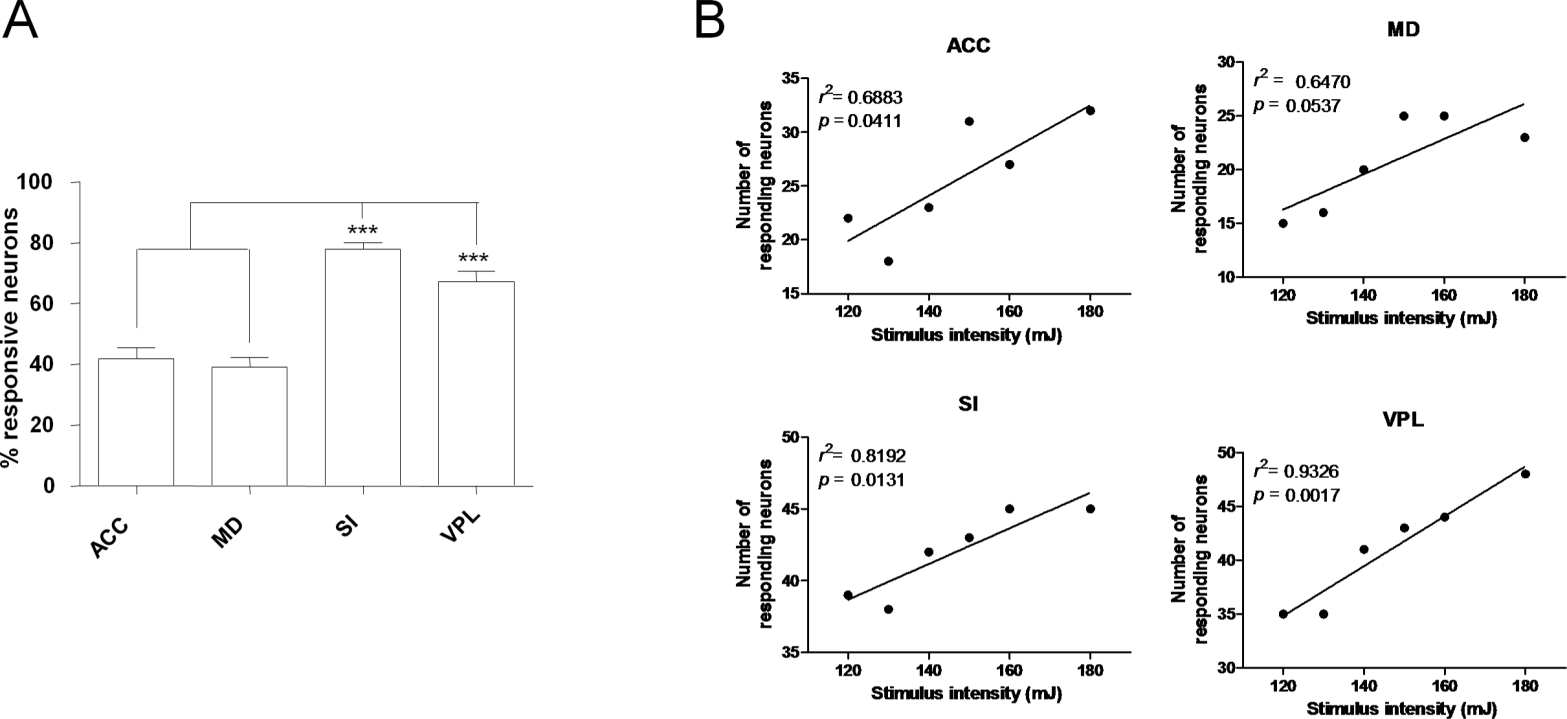
**Neuron number coding of stimulus intensity**. (A) According to the neuron number coding scheme, there is a higher percentage of laser-responsive neurons in the lateral pathway (SI and VPL) than in the medial pathway (ACC and MD). ***p < 0.001, data are means ± SEM. (B) The relationship between responsive neuron number and stimulus intensity. Significant (ACC, SI, and VPL), or near significant (MD), positive correlations were found in all of the recorded areas.

Table 1 shows the number and percentage of neurons that were activated in response to different intensities of laser stimulation. For each brain region studied, the number of responsive neurons increased with stimulus intensity. One-way ANOVA demonstrated an effect of brain region on percentage of responsive neurons. The subsequent post hoc test revealed a significant difference between regions of the medial and lateral pathways (Figure 4A), with a higher percentage of laser-responsive neurons being encountered in the lateral pathway (i.e., SI and VPL) than in the medial pathway (i.e., ACC and MD) (*p *< 0.001). Our correlation analysis revealed positive correlations between the number of active neurons and stimulus intensity in each of the recorded areas (for ACC, *r^2 ^*= 0.6883, *p *= 0.0411; for MD, *r^2 ^*= 0.6470, *p *= 0.0537; for SI, *r^2 ^*= 0.8192, *p *= 0.0131; for VPL, *r^2 ^*= 0.9326, *p *= 0.0017; see Figure 4B). It should be noted that the correlation coefficients between neuron number and stimulus intensity were higher in the lateral pathway than in the medial pathway (*r^2 ^*= 0.8192 & 0.9326 vs. *r^2 ^*= 0.6883 & 0.6470), suggesting that stimulus intensity coding differs between the neurons in these two pathways.

**Table 1:** Number of neurons responding to stimuli of different intensities

Brain area	Laser energy intensity (mJ)						Total
		
	120	130	140	150	160	180	
ACC	22 (36.1%)	18 (29.6%)	23 (37.7%)	31 (50.8%)	27 (44.3%)	32 (52.5%)	61
MD	15 (28.3%)	16 (30.2%)	20 (37.7%)	25 (47.2%)	25 (47.2%)	23 (43.4%)	53
SI	39 (72.2%)	38 (70.4%)	42 (77.8%)	43 (79.6%)	45 (83.3%)	45 (83.3%)	54
VPL	35 (57.4%)	35 (57.4%)	41 (67.2%)	43 (70.5%)	44 (72.1%)	48 (78.7%)	61

#### Coding by firing rate

**Figure 5:**
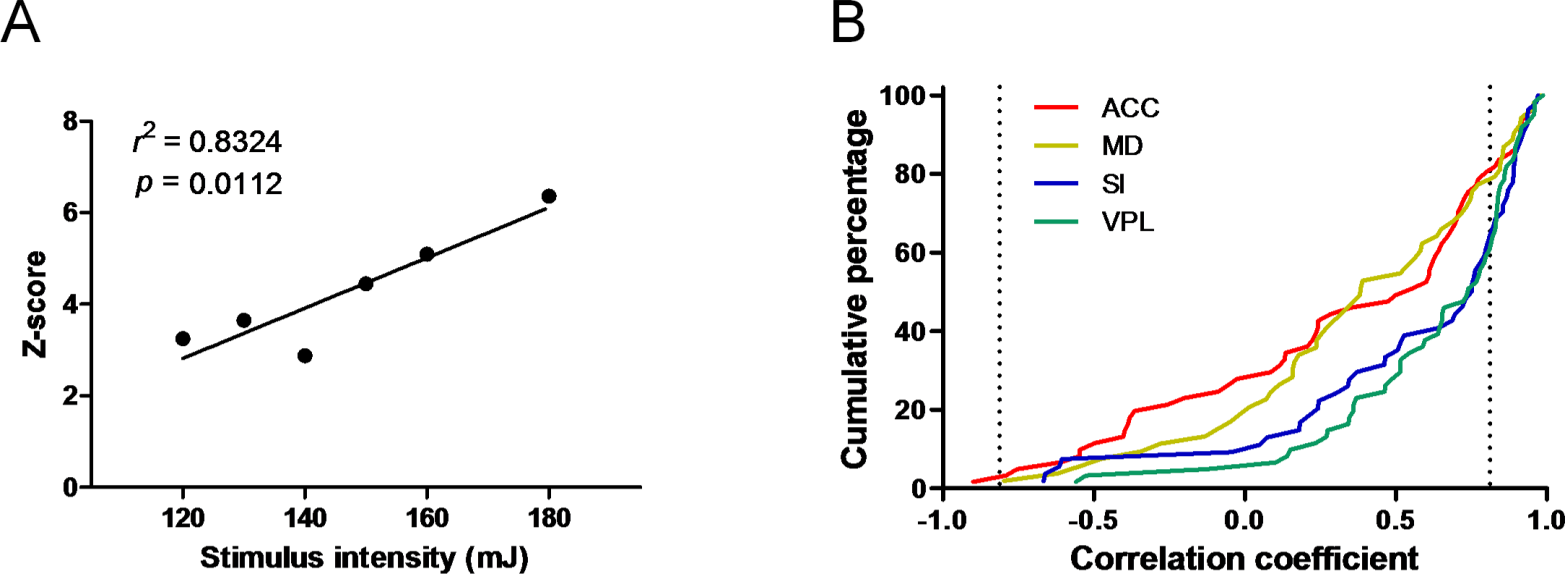
**Wide-dynamic coding of stimulus intensity**. (A) An example of a WDR neuron that showed positive correlation between normalized firing rate (Z-score) and stimulus intensity. (B) The cumulative distribution of correlation coefficients (*Z*-score vs. laser intensity). The dotted lines indicate the upper and lower limits, respectively, of the normal range. Correlation coefficients that lie outside these limits were considered significant. Thus, the cumulative percentage outside the limits represents the proportion of WDR neurons for each region.

Because stimulus intensity is often considered to be coded in the firing rate of neurons, we examined the correlation between laser intensity and firing rates of the simultaneously recorded neurons. For each brain recording area, linear regression analysis was performed between the two variables and revealed a significant positive correlation in more than one fifth of the neurons. In accordance with the conventional classification scheme, we defined those neurons that exhibited increased firing rates in response to increasingly intense stimuli as wide dynamic range (WDR) neurons. Figure 5A illustrates an example of a WDR neuron that increased its discharge frequency with increasing stimulus intensity (*r^2 ^*= 0.8324, *p *= 0.0112). The cumulative percent distribution of correlation coefficients is shown in Figure 5B.

As shown in Table 2, *Chi*-square tests revealed that the proportion of WDR neurons in the total number of neurons was higher in the VPL of the lateral pathway than in the ACC of the medial pathway (*p *= 0.0481), trended toward being higher in the VPL than in the MD of the medial pathway (*p *= 0.0699), and trended toward being higher in the SI of the lateral pathway than in the ACC (*p *= 0.0678). By contrast, the number of WDR neurons calculated as a percent of the number of nociceptive-responsive neurons did not differ between the two pathways (*p*'s > 0.05). These results suggest that neurons in the lateral and medial pathways may share the responsibility for encoding noxious stimulus intensity.

**Table 2:** Ratios of WDR neurons in each brain region of interest

Brain area	WDR/ total neurons	*p *value		WDR/responsive neurons	*p *value	
				
		vs. ACC	vs. MD		vs. ACC	vs. MD
ACC	13/61 (21.3%)			13/26 (50.0%)		
MD	12/53 (22.6%)			12/21 (57.1%)		
SI	20/54 (37.0%)	0.0678	0.1396	20/42 (47.6%)	1.0000	0.5950
VPL	24/61 (39.3%)	0.0481	0.0699	24/48 (50.0%)	1.0000	0.6116

**Figure 6:**
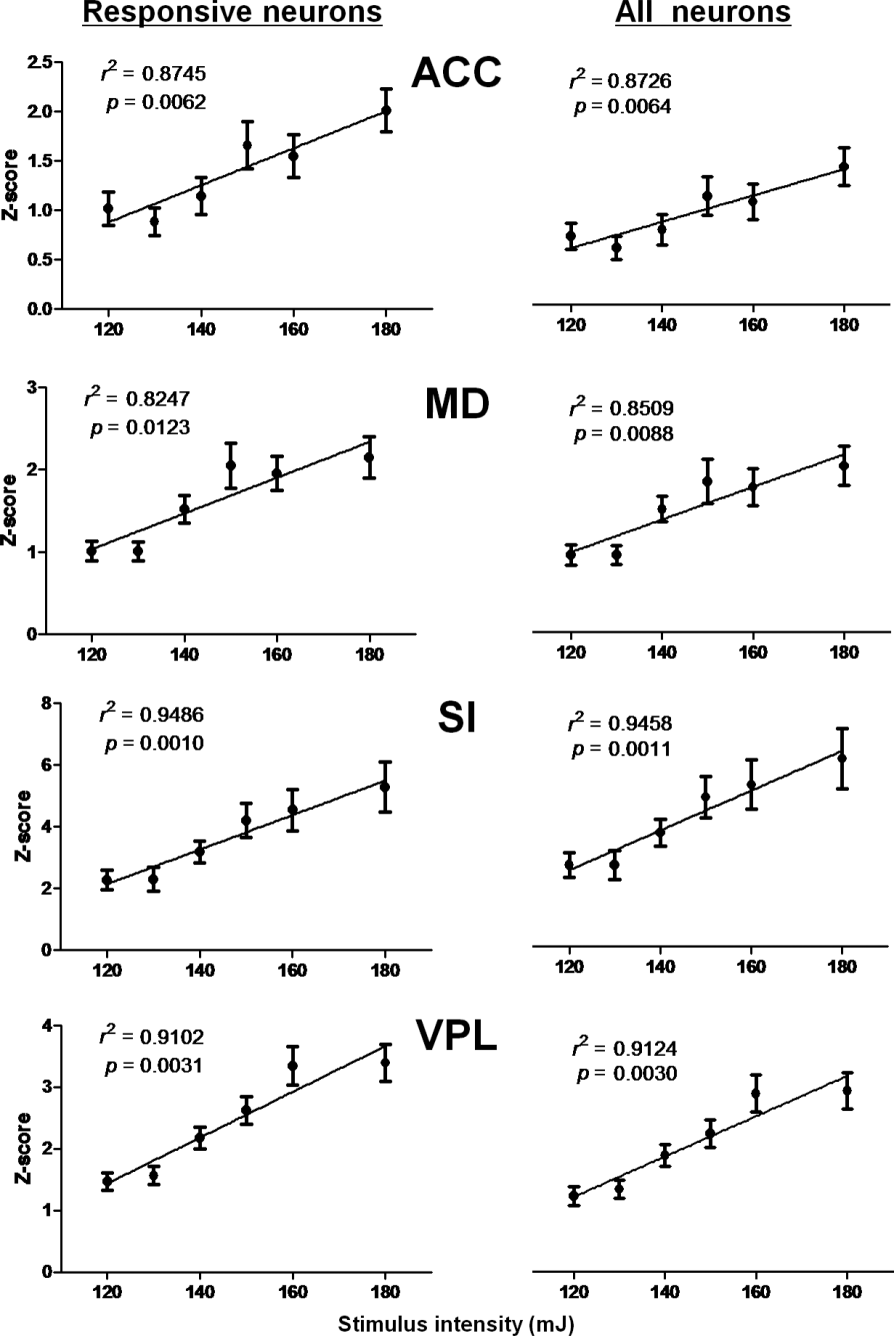
**Normalized firing rates of responsive neurons (left column) and of all recorded neurons (right column) correlated with stimulus intensity**. The Z-scores were averaged across all neurons within each population.

In order to clarify whether the encoding of noxious stimulus intensity requires all neurons or simply the reactive neurons, we further investigated the correlation between the mean *Z*-scores for firing rate and stimulus intensity using different sampling schemes (Figure 6). All four brain regions showed strong positive correlations in both the all-neuron and the responsive-neuron conditions. No remarkable differences were observed in any of the regions with respect to the degree of linear relationship between the two sampling schemes (ACC, *r^2 ^*= 0.8745, *p *= 0.0062 vs. *r^2 ^*= 0.8726, *p *= 0.0064; MD, *r^2 ^*= 0.8247, *p *= 0.0123 vs. *r^2 ^*= 0.8509, *p *= 0.0088; SI, *r^2 ^*= 0.9486, *p *= 0.0010 vs. *r^2 ^*= 0.9458, *p *= 0.0011; VPL, *r^2 ^*= 0.9102, *p *= 0.0031 vs. *r^2 ^*= 0.9124, *p *= 0.0030), suggesting that all neurons, including both responsive and non-responsive neurons, contribute to the encoding of noxious stimulus intensity.

#### Mass spike count coding

**Figure 7:**
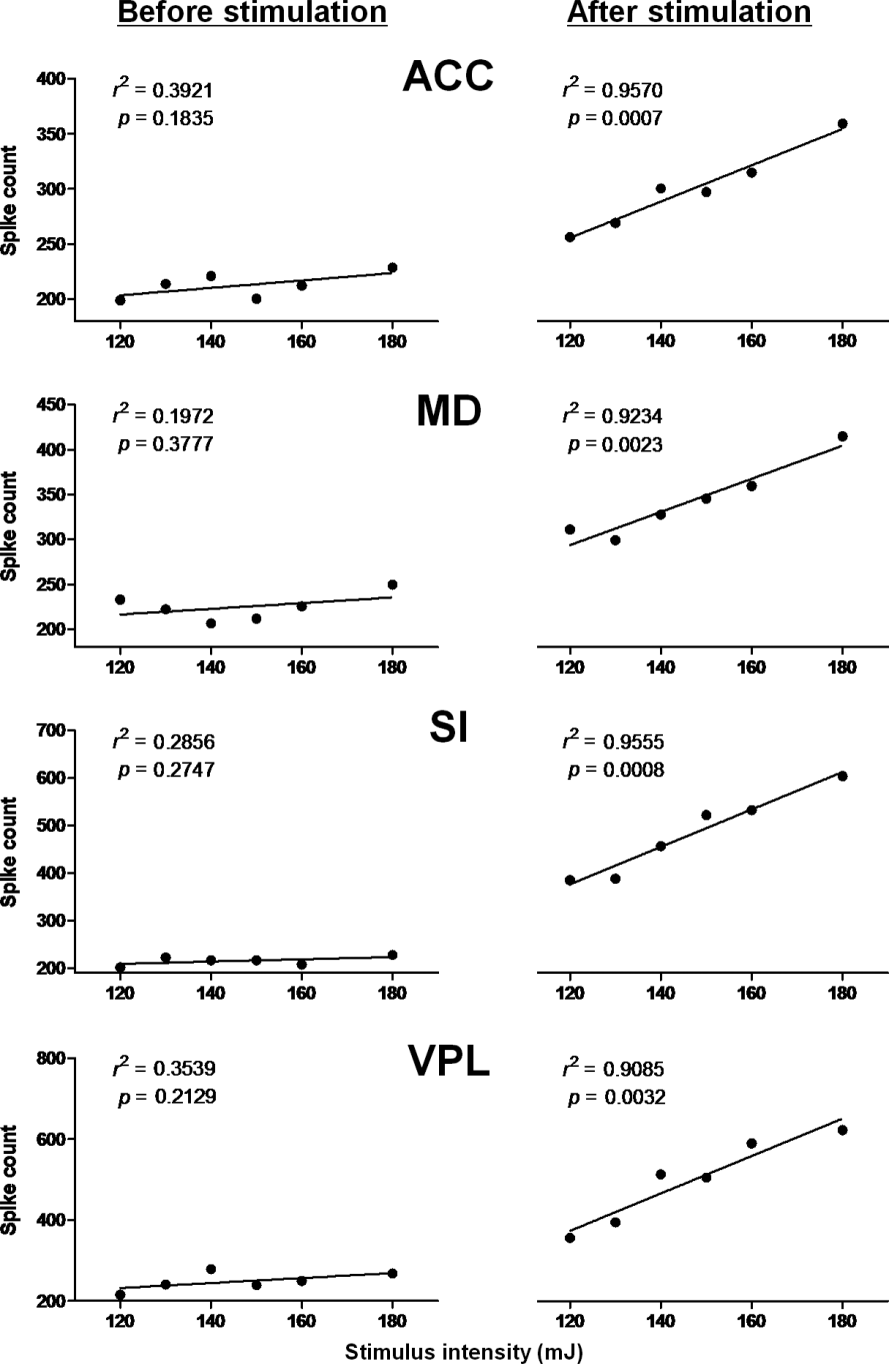
**MSCs correlations between stimulus intensity and mass spike counts before (left column) and after laser stimulation (right column)**. Significant positive correlations were observed following the application of noxious laser energy.

It is possible that both the number of active neurons and their firing rates together encode information about the intensity of the pain stimulus. Therefore, we calculated the mass spike count (MSC), and examined the correlation between the MSC of 0 - 1 sec after laser stimuli and the stimulus intensity in each region. The MSCs correlated strongly with stimulus intensity (Figure 7), and the degree of correlation using MSC coding was generally higher than that with either frequency coding (see Figure 6) or number coding (see Figure 4B). No differences were found among regions in the medial and lateral pathways with regards to MSC coding (ACC, MD vs. SI, VPL: *r^2 ^*= 0.9570 and 0.9234 vs. *r^2 ^*= 0.9555 and 0.9085). These results suggest that neurons in the two parallel pain pathways may both play important roles in coding stimulus intensity.

### Intensity coding at the neural ensemble level

**Figure 8:**
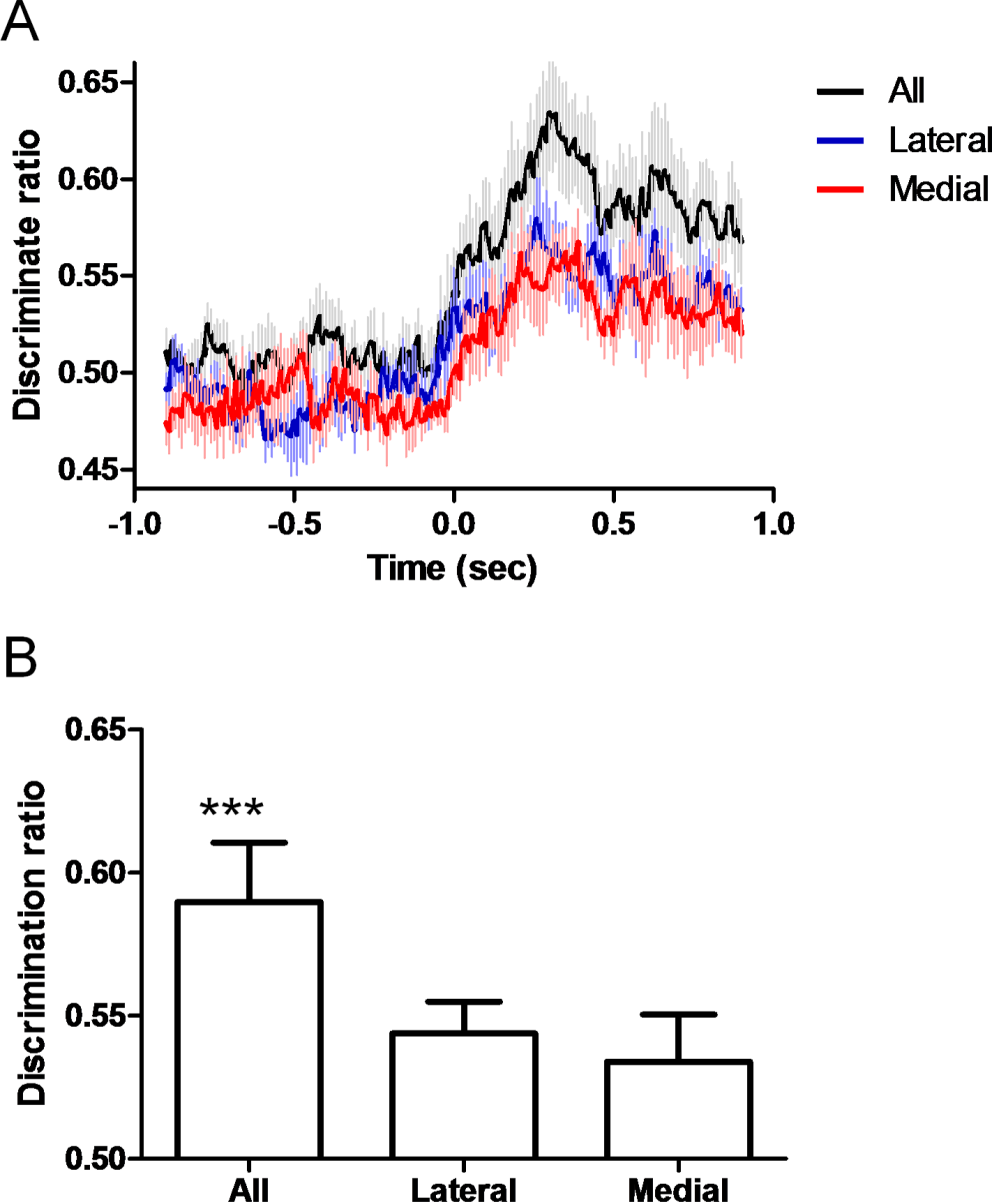
**The capacity of neural ensemble discrimination of different stimulus intensities measured by discriminant analysis**. The discrimination capacity of total neurons recorded was significantly better than that of neurons recorded in either pathway alone. No difference was found between the medial and lateral pathways. (A) The temporal distribution of discrimination ratio from 1.0 s before to 1.0 s after stimulation. The "leave-one-out" method was used to calculate the discrimination ratio (see Methods). (B) Statistical comparison of the discrimination performance among the whole network, in the medial pathway only, and in the lateral pathway only. ***p < 0.001. All: all the neurons. Lateral: neurons in the lateral pathway. Medial: neurons in the medial pathway. Data are presented as means ± SEM.

Because sensory information is considered to be best represented by neural populations, we further investigated stimulus intensity coding at the neural ensemble level. The capacity of neural ensembles to discriminate between different stimulus intensities was evaluated by measures of discriminant analysis. The percentage of correct discriminations was calculated and compared among the following neural populations: all the recorded neurons, those within the medial pathway, and those within the lateral pathway (Figure 8A). As expected, the discrimination capacity was significantly better when all of the neurons were used than when neurons of either pathway were used (Figure 8B). No difference was found between the parallel pathways, confirming that they were of equal importance for intensity encoding.

### Histological localization of recording sites

**Figure 9:**
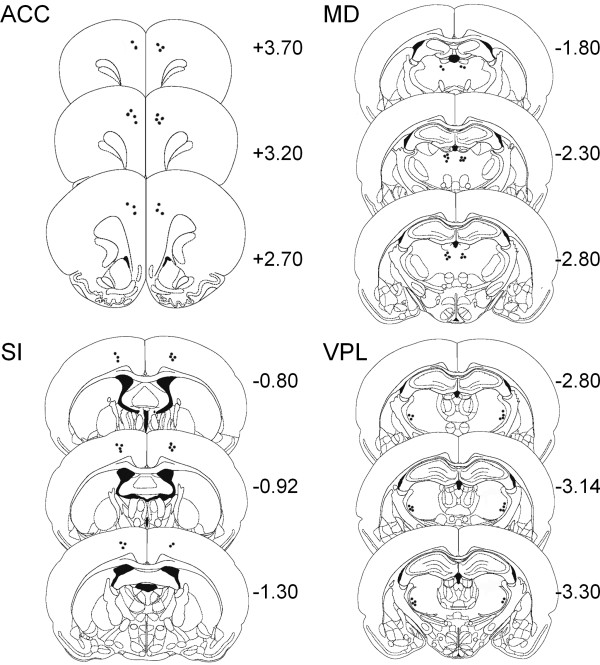
**Schematic drawing indicating the locations of recording electrodes in the ACC, SI, MD, and VP**. The black dots indicate the positions of iron deposits at the tips of the selected microwires. Numbers on the right side of each panel indicate the distance rostral (+) or caudal (-) to Bregma in mm.

The locations of the recording electrodes from which data are reported in this study are shown in Figure 9. Within the cingulate cortex, most of the iron deposits were found in the anterior areas. In the somatosensory cortex, most of the recording tips were in the hind limb region. In the thalamus, the tips were primarily located in the mediodorsal and ventroposterior parts of the thalamus. Data obtained from electrode tips found to be outside the designated areas were not included in the analysis.

## Discussion

In the present study, we investigated how the intensity of pain stimuli is encoded by neural ensembles within central sensory and affective pain pathways. We used a multichannel single-unit recording technique to measure neuronal activity within the SI, VPL, ACC, and MD in response to laser-evoked pain in the rat paw. The main findings of the present work were that the noxious laser stimulation evoked uniform double-peak responses in all of the recorded brain regions. The coding capability of the medial (affective) pathway neurons for noxious stimulus intensity can be comparable to that of the lateral (sensory) pathway, demonstrated by various analysis methods including the neuron number coding, firing rate coding, as well as mass spike count (MSC) coding. Furthermore, our data indicate that the MSCs best represent the coding strategy of neurons for the noxious intensity differentiation in the parallel pain pathways.

### Laser- evoked double-peak responses in the lateral and medial pathway neurons

The present study showed that the laser stimulus evoked various types of single-unit responses in the four recorded regions, including single-peak, double-peak, brief, as well as long-lasting neuronal activity. However, computing the mean firing rate revealed uniform double-peak responses in all the brain regions with short latency (< 100 ms) and long latency (300-500 ms). The results were consistent with previous studies by Kuo and Yen in which they observed short- and long- latency laser-evoked cortical responses in the rat primary sensorimotor cortex (SmI) and ACC [8]. The laser-evoked early and late peaks have been generally thought to coincide with the perception of the first (fast or pricking) and second (slow or burning) pain, respectively, since the laser beam could selectively and synchronously activate cutaneous thermonociceptors which conduct in the Aδ and C fiber ranges [10].

The SI has been reported to be involved in the C-fiber-mediated nociceptive processing in different animal models. Altered sensory processing related to hyperalgesia has been reflected in the C-fiber laser-evoked potentials in the rat SI cortex [11]. By recording the nociceptive C-fiber evoked potentials from the surface of SI, Kalliomäki et al. demonstrated that following laser stimuli there are at least two different C fiber projections to SI, one somatotopically organized, and one more diffused organized input. Moreover, the spinal NMDA-receptor mechanisms may amplify the acute transmission of nociceptive C fiber input to SI in a frequency-dependent way [12,13].

The experimental data is rare about the activation of medial pain system by the A-fibers. However, in a very recent study, by means of retrograde transneuronal transport of rabies virus, the electrophysiological evoked responses in ACC were recorded with such short latencies (less than 20 ms) [14]. They also demonstrated that ascending somatosensory pathways to the ACC most likely originated from the myelinated Aδ and Aβ fibers but not from unmyelinated C fibers.

### The coding capacity for noxious stimulus intensity in the lateral and medial pain pathways

It has long been believed that the somatosensory cortex and sensory thalamic nuclei are responsible for the sensory discrimination of pain, including discriminant of the intensity, quality, duration, and location of painful stimuli [15-17]. In an early study investigating the thalamic neuronal response to noxious stimuli in macaque monkeys, Kenshalo et al. found that the discharge frequency of VPL neurons increased progressively in response to presentation of graded noxious heat stimuli [18]. Exploring the effect of laser intensity on evoked potentials in several brain regions in surgical patients, including the primary somatosensory cortex, Ohara et al. showed that the peak amplitudes of both early (N2) and late (P2) pain-related components correlated with laser energy intensity [19]. In the present study, we found that the mean firing rates, the number of responsive neurons, and the total spike counts in all of the four recorded regions constituting the pain network increased with laser stimulus intensity. Significant correlations were observed between these parameters and laser energy, suggesting the involvement of parallel pain pathways in the processing of noxious intensity. Thus, our results extend the previous findings to the single-unit level and underscore the importance of the sensory thalamus and cortex in coding noxious stimulus intensity.

On the other hand, in contrast to the convergence of data reported for the lateral pain pathway, the role of medial pathway neurons in encoding noxious intensity is still controversial. Several brain imaging studies from about a decade ago did not support a role for the ACC in coding pain intensity. Neither regional blood flow nor blood oxygenation level-dependent signal in the ACC correlated with noxious stimulus intensity [20-22]. Those imaging studies in human subjects conflicted with findings from animal studies using electrophysiological recording in monkeys and rabbits that provided evidence for the involvement of the ACC and the medial thalamus in noxious intensity coding [23-26]. More recently, an fMRI study using human subjects demonstrated pain intensity-related activation of the ACC and the dorsomedial and centromedian thalamus [6]. These findings challenge the traditional view that the sensory-discriminative processing of pain is confined to the sensory pathway. In the current study, both neuronal and ensemble activation responses support a role of the medial pain pathway in pain intensity coding. The single-unit analysis of medial pathway neurons in the present study showed that firing rates, number of responsive neurons, and MSCs correlated with noxious stimulus intensity. Moreover, our ensemble data suggested that the medial and lateral pain systems may both be important for intensity discrimination. These results expand our understanding of pain perception by highlighting a role of the medial pain pathway.

### Noxious stimulus intensity is best encoded by mass spike counts

Three kinds of coding methods were investigated in this study: neuron number coding, firing rate coding, and MSC coding. The former two are classic methods that have been widely used in sensory-related encoding studies [27-29]. The proportions of WDR neurons in the total sample of neurons in the SI and VPL were higher than those in the ACC and MD, suggesting that the sensory pathway may better encode nociceptive stimulus intensity than the affective pathway. This result seems to agree with the traditional view. For instance, when Kuo and Yen compared the neuronal responses to noxious laser-heat stimuli in the ACC and SmI, they found that ACC neurons were less responsive than SmI neurons [8].

On the other hand, we did not detect differences in coding efficiency between the medial and lateral pathways when MSC coding was used. This result is consistent with our discriminant analysis results. The correlation coefficients using spike count coding were obviously greater than those obtained when either frequency or number of coding neurons were used, indicating that the MSC coding strategy represented stimulus intensity better than the other two did. Because spike count analysis considers neuronal number and frequency coding simultaneously, there is no loss of information. Recent studies have shown that spike count is very useful, and more efficient and available, for neuronal encoding [30-33]. Luna et al. tested five possible candidate codes, including inter-spike intervals, mean spiking rate, mean burst rate, and absolute number of spikes or bursts, to determine the optimal neural code for discriminating two consecutive vibrotactile stimuli. They found that only the spike count code was consistent with the psychophysical measures [32]. Another recent study successfully used the spike count code to find the best time scale for predicting monkeys' choices in a speed change detection and discrimination task [33]. One might argue that the spike count code is, in essence, the same as the firing rate code. However, a normalized firing rate, such as that used in this study, does not take baseline neuronal activity into consideration, and thus may interfere with the assessment of coding capability. On the other hand, this result also indicates that the neuronal ensemble may represent the painful stimuli with a random/alternative manner, in which some neurons of the ensemble respond in one trial while other neurons in another trial. While keeping the MSC constant which bring about a steady representation of the stimulus intensity, this coding method has the advantage of giving neurons a chance to relax and restore the energy consumed. This will be extremely useful when dealing with repeated stimulation, as in the case of our current experimental protocol. Therefore, our results suggest that a strategy based on the MSC may be the best neural code for the central nervous system to use to discriminate different intensities of sensory stimuli.

## Conclusion

In the present study, we analyzed the central coding strategies of nociceptive stimulus intensity within the parallel pain pathways at both single neuron and neural ensemble levels, and compared three types of coding schemes, i.e., neuron number coding, firing rate coding and mass spike count coding. We found significant correlations between laser intensity and the number of responsive neurons, the firing rates, as well as the mass spike counts (MSCs). The coding capacities of medial and lateral pain pathway neurons were comparable. In addition, the degree of correlation using MSC coding was higher than that with either frequency coding or number coding, suggesting that the MSC coding may be a more reliable method than neuron number coding or firing rate coding for elucidating nociceptive information transmission and processing in the brain.

## Methods

### Animals and surgical procedures

Eight awake adult male Sprague-Dawley rats, weighing 250-300 g, were used. Animals were housed individually, with *ad libitum *access to food and water, under a reverse 12:12 light/dark cycle (the light phase commenced at 8:00 am). All experiments were carried out in accordance with the National Institutes of Health Guide for the Care and Use of Laboratory Animals (U.S.A.). The research protocol was approved by the Institutional Animal Care and Use Committee of the Chinese Academy of Sciences. The protocol was designed to minimize pain and distress and the number of animals used in the study.

Four microelectrode arrays were implanted in the brain by making four small craniotomies. Animals were anesthetized with sodium pentobarbital (50 mg/kg, i.p.) and then mounted on a Kopf stereotaxic apparatus (David Kopf Instruments, USA). Supplementary doses of anesthetic (one third of the original dose) were given when necessary. Coordinates for the craniotomies were determined according to a rat brain atlas [34]. ACC was 3.2 mm rostral to Bregma (3.2 A), 0.8 mm lateral (L) to midline and 2.8 mm ventral (V) to the surface of the skull. SI was 1.0 mm caudal to Bregma (-1.0 A), 2.0 L and 2.0 V. MD thalamus was -2.3 A, 0.8 L and 5.5 V. VPL thalamus was -3.0 A, 3.0 L and 6.0 V. Four bundles of eight stainless steel Teflon-insulated microwires (50-μm diameter, Biographics, Winston-Salem, NC) were slowly lowered into the unilateral target areas. The microwires were connected to pins on the headset which was secured to the cranium with dental cement using skull screws as anchors. Ground wires were securely attached to the skull screws. Animals received penicillin (60,000 U, i.p.) before surgery and were housed individually in cages for 7 days while they were recovering from the surgical procedure.

Prior to the experiment, each animal was handled daily and habituated to a transparent plastic recording chamber (40 × 40 × 30 cm^3^) for 7 d. On each of the recording days, rats were allowed a 30-min period to become familiar with the chamber.

### Laser stimulation

Stimuli (wavelength, 10.6 μm; beam diameter, 2.5 mm; pulse duration, 20 ms) were generated from a CO_2 _laser stimulator (Institute of Optical Precise Engineering and Physics, Chinese Academy of Sciences, China) in single-pulse mode [8,9]. The CO_2 _laser beam projection was guided by a helium laser producing a red light spot. Laser pulses were applied to the plantar surface of the hind paw contralateral to the brain recording sites. Neuronal activity and observations of nocifensive behaviors (paw withdrawal or licking) were recorded. Each recording session consisted of about 40 trials with the laser pulses delivered at the same energy level. To minimize tissue damage, sensitization, and habituation, the stimulation site was varied and the interstimulus interval was ≥ 40 s. A total of 6 sessions were given. In each session the output energy of the laser was increased to a higher level (120, 130, 140, 150, 160 and 180 mJ). The laser pulse at the lowest intensity was perceived as a mildly painful sensation when applied to the experimenter's skin.

### Electrophysiology

Neuronal activities were recorded extracellularly via stainless steel microwires and the signals were passed from the headset assemblies to a preamplifier. The signals were band-pass filtered between 0.5 and 5 kHz (6 dB cutoffs) before being sent to a multichannel spike-sorting device. Spike activities were monitored by a computer with a 20-μs time resolution and identified by setting proper boxes for amplitude and duration with a PC-based software *Magnet *(Biographics, Inc). The identity of a clearly sorted single neuron was verified by graphical capture of action potential waveforms which were inspected continuously to ensure that the same neuron was recorded during each experimental recording session. Waveform time stamps were stored in a database for off-line analysis with the commercially available PC-based programs *Stranger *(Biographics Inc., USA), *Neuroexplorer *(Plexon Inc., Dallas, TX, USA), *Matlab *(MathWorks Inc. USA), and *Prism *(GraphPad Software, Inc., USA). Behavioral responses were also recorded during each session using *Magnet *software and a video camera (Vigour, VG-2012, China).

### Data analysis

#### Nocifensive behavior

Data were expressed as the percentage of paw withdrawal or licking, which were calculated according to the following formula: (number of stimulations producing paw withdrawal or licking/total number of stimulations) × 100%. The data were then analyzed by one-way analysis of variance (ANOVA), followed by a linear trend analysis across stimulus intensities. The accepted level of statistical significance was *p *< 0.05.

#### Electrophysiological data

Bin counts for each trial were calculated using the analysis program *Neuroexplorer*. A 10-ms bin size was used for the computation of peri-stimulation time histograms (PSTHs) with a time range of -1.0 to +1.0 s relative to the stimulus onset. The firing rates for each unit and each time bin were then transferred into *Z*-scores: *Z *= (*X *- *M*)/*S*, where *X *is the actual firing rate obtained from PSTH, and *M *and *S *are the mean and standard deviation of the basal neuronal firing rate. An increase or decrease in firing rate that was more than two standard deviations from the baseline (-1 - 0 s) for at least three consecutive bins was considered to be an excitatory or inhibitory response. A cluster technique (K-means, SPSS) was used to sort neuronal responses based on the similarities in patterns of excitation or inhibition around stimulation events. *Z*-scores were arranged in clusters to visualize the response pattern of neuronal populations.

The number of neurons exhibiting an excitatory or inhibitory response to the laser stimulation (i.e., responsive neurons) was counted. *Chi*-square test was employed to detect significant differences in the number of responsive neurons between brain regions. One-way ANOVA followed by the *Newman-Keuls *post hoc test was used to compare the averaged percentages across six intensities between the four recorded brain regions. Linear regression analysis was performed to assess the relationship between variables (i.e., firing rates, number of responsive neurons, or mass spike count) and stimulus intensity. The firing rates were transformed to *Z*-scores as described above. The cumulative frequency distribution of correlation coefficients (*Z*-score vs. laser intensity) were calculated and displayed as percentages. The mass spike count (MSC) was the summation of spikes emitted by all single units in a trial within a 1-s poststimulus time window. Thus,

$$MSC=\overset{N}{\underset{i=1}{\sum }}C_{i},$$

where *N *is the number of single units in a given brain area, and *C_i _*is the spike counts of the *i*th single unit in that area during a given time epoch around the stimulation.

#### Discriminant analysis

A linear discrimination method was employed in the analysis. Firstly, principal component analysis (PCA) was performed for all neurons recorded in each rat. The first twelve principal components (PC1 to PC12) with 10-ms bin sizes for data from -1 s to 1 s around the stimulus were exported to MATLAB. A moving-window technique was used with a 20-bin window (200 ms) and a 1-bin step (10 ms) to calculate the mean component scores. A leave-one-out cross-validation procedure, in which a kernel *Fisher *discriminant classifier was constructed excluding a single trial from the data which was then used to test the classifier, was performed to estimate the correct rates for each data set. This procedure was repeated N times (N is the number of the total trials) and the results were pooled to estimate the general correct rate (R *_all_*). Secondly, the firing rates of all the recorded neurons in the medial/lateral pathway were set to zero to get R *_-m_*/R *_-l_*, which is the correct rate of discrimination without the contribution from neurons in the medial/lateral pathway. The contribution of the medial/lateral pathway neurons to the discriminant analysis (*R_m _*and *R_l_*) was then be calculated as:

$$R_{m}=R_{all}-R_{-m}\;\text{and }R_{l}=R_{all}-R_{-l}$$

The means and standard errors of these contributions from all rats were computed over time. Then the mean values of *R_all_*, *R_m _*and *R_l _*from 0 to 1 s post-stimulation were compared using one-way ANOVA.

### Histology

After completion of the stimulation and recording sessions, rats received an overdose of sodium pentobarbital. Recording sites were marked by electrophoretically depositing iron (20 μA DC current, 20 s duration, and anode at the electrode) at the tips of selected wires. Animals were perfused with 4% paraformaldehyde and 5% potassium ferricyanide solution. The brains were post-fixed in a solution of 20% sucrose followed by a solution of 30% sucrose and then stored at 4°C until time of sectioning. Forty-micron-thick coronal sections were cut through the ACC, SI, and the MD and VP thalamus. The slides were stained with hematoxylin and eosin (H-E). Recording sites were determined under a light microscope. The iron deposits were easily identified as blue dots. Data from sites found to be outside the target areas were removed from the overall analysis.

## List of abbreviations

ACC: Anterior cingulate cortex; MD: Medial dorsal thalamus; SI: primary somatosensory cortex; VPL: ventral posterior lateral thalamic nucleus; PCA: principal component analysis; PSTH: peri-stimulation time histograms; SmI: primary sensorimotor cortex; WDR: wide dynamic range; MSC: Mass spike count; fMRI: functional magnetic resonance imaging; LEP: laser-evoked potential.

## Competing interests

The authors declare that they have no competing interests.

## Authors' contributions

YZ carried out all the experiment and performed the statistical analysis. NW assisted with the electrophysiological recordings and data analysis. JYW participated in the design of the study and drafted the manuscript. JYC and DJW helped in the methodology of electrophysiological recording. FL conceived of the study, and participated in its design and helped to draft the manuscript. All authors read and approved the final manuscript.

## Authors' information

FL is currently the Professor and Director of the Key Laboratory of Mental Health in the Institute of Psychology, Chinese Academy of Sciences; a committee member of the Chinese Association for the Study of Pain; first started the multichannel single-unit study of pain in behaving animals in China. JYW is the Associate Professor of the IPCAS, the first author of the earliest papers on ensemble neuronal pain responses in the thalamocortical pathways in behaving animals. YZ is a PhD student and NW a postdoc of FL. DJW is the Director of the Neuroscience Research Institute of North Carolina, one of the founders of the multiple channel single-unit recording technique in awake animals. JYC is the Associate Professor of the NRINC, who helped FL to establish the electrophysiological lab in Beijing.
